# 

**Published:** 1920-05-29

**Authors:** 


### A Medical Centenarian.

The congratulations and good wishes of all will be extended to Dr. J. S. S. Logie, of Orkney, who has now celebrated his hundredth birthday. In addition to being a medical centenarian, Dr. Logie is also stated to be the oldest elder of the Scottish Church.

### Matrons' and Sisters' Appointments.

Romford Union Infirmary.—Miss Mary Ellen Williams, Miss Eva Mary Snow, and Miss Elizabeth Ellen Robinson have been appointed sister matron, deputy sister matron, and charge sister respectively. Miss Williams was trained at West Ham Infirmary, and has since been superintendent nurse at the Guardians' Institution, Tunstall, and at the Union Infirmary, Worcester. She holds the certificate of the Central Midwives Board. Miss Snow was trained at Lewisham Infirmary, has been charge sister at Kensington Infirmary, and has done Army nursing; she holds the certificate of the Central Midwives Board and the Royal Sanitary Institute Health Visitor's certificate. Miss Robinson was trained at the Romford Union Infirmary.

East Suffolk and Ipswich Hospital.—Miss Alice A. R. Bunch has been appointed matron. She was trained at Burton-on-Trent General Hospital, and has since been charge sister at Brompton Hospital and Birmingham General Hospital and the 1st Southern General Hospital, Birmingham. She has also been assistant matron of St. Mark's Hospital, City Road, E.C.

Royal Victoria and West Hants Hospital, Boscombe.—Miss Betty Walker has been appointed matron. She was trained at Lambeth Infirmary, and served in Queen Alexandra's Imperial Military Nursing Service Reserve as matron of Prees Heath Military Hospital. She has also been sister and matron's assistant at Bristol Royal Infirmary and matron of Warminster Cottage Hospital, and of Parc Wern Auxiliary Hospital, Swansea. Miss Walker was mentioned in despatches and awarded the Royal Red Cross, First Class, during the war.

North Eastern Fever Hospital, South Tottenham.—Miss Jeanette Gray Lamb has been appointed sister. She was trained at Perth Royal Infirmary and Glasgow City Fever Hospital.

County Infirmary, Newtown, Montgomery.—Miss Ethel Jones has been appointed sister. She was trained at Hackney Union Infirmary, and has since been staff nurse and temporary sister at the Norfolk and Norwich War Hospital, and sister at the Cottage Hospital, Oswestry.

Northern Sanatorium and Convalescent Fever Hospital, Winchmore Hill.—Miss A. M. Bisset, Miss E. MacDonald, and Miss H. G. Ballard have been appointed sisters. Miss Bisset was trained at Shoreditch Infirmary, and has since been staff nurse in Queen Alexandra's Imperial Military Nursing Service Reserve, serving both at home and abroad. Miss MacDonald was trained at Mill Road Infirmary, Liverpool, where she was later sister; she has also been sister at the Isolation Hospital, Southampton, the Harton Hospital, South Shields, the Southern Hospital, Dartford, and in Queen Alexandra's Imperial Military Nursing Service Reserve at Ripon Military Hospital. Miss Ballard was trained at the City of Westminster Infirmary, Hendon, and has since been sister in the Territorial Force Nursing Service, serving at home and in France.

### The College of Nursing
### (Limited by Guarantee).

BADGES RETURNED THROUGH THE POST.

College Badges issued by Messrs. Gaunt and Sons bearing the following registration numbers have been returned by the Post Office to the College: 15,384, 12,872, 13,350, 9,233, 7,339, 2,883, 11,165. Members registered under these numbers are asked to communicate with the Registrar of the College of Nursing, Ltd., 7 Henrietta Street, Cavendish Square, London, W. 1, giving their present permanent address, when the Badges will be forwarded.

LONDON CENTRE.

The London Centre Club was "at home" to about two hundred College members at 7 Henrietta Street on Thursday evening, May 20. The Musical Society of St. Thomas's Hospital, composed of members of the nursing staff, provided a delightful programme which was very much enjoyed by everyone. A telegram was read from Dame Sidney Browne expressing regret that she was unable to be present. Miss Gibson thanked the performers for the pleasure they had given, commented on the exceptional talent they had displayed, and put into words what was made evident that evening, namely, that the Centre had already outgrown the limited accommodation possible at headquarters, and that it must at once seek more extensive premises. It was evident that the members agreed with these remarks by the applause which followed.

BIRMINGHAM CENTRE.

A meeting was held in the lecture theatre of the General Hospital, Birmingham (Miss Musson in the chair), on May 19, at 5.30 p.m., when the scheme to found a London Club for all members of the College came up for discussion. A fair proportion of the members present were in favour of the scheme, but the general opinion was strongly non-committal until more concrete proposals were forthcoming.

A "THREE COUNTIES" CLUB.

It is hoped next year, by means of a large scenic fair, to raise sufficient funds to establish in Birmingham a club for nurses in the three counties, and also to contribute a substantial sum towards the Endowment Fund of the College and the Tribute Fund for Disabled Nurses. Mrs. H. S. Richards, who has kindly consented to act as organising secretary of the Fair, briefly outlined the scheme. At the conclusion of the meeting a vote of thanks was unanimously accorded her.

By special request a post-graduate lecture on "Tuberculosis" will be given in June, the name of the lecturer and the date of the lecture to be announced later.

### Estates of Medical Men.

Mr. Thomas Pickering Pick, F.R.C.S., the eminent surgeon, left the MS. lectures of John Hunter and a portrait of himself by Phil. Morris, R.A., to the Royal Academy of Medicine.

